# Analysis of Thermal Stress in Vanadium Dioxide Thin Films by Finite Element Method

**DOI:** 10.3390/nano12234262

**Published:** 2022-11-30

**Authors:** Yuemin Wang, Lebin Wang, Jinxin Gu, Xiangqiao Yan, Jiarui Lu, Shuliang Dou, Yao Li, Lei Wang

**Affiliations:** 1 Shenzhen Key Laboratory of Polymer Science and Technology, College of Materials Science and Engineering, Shenzhen University, Shenzhen 518060, China; 2 College of Physics and Optoelectronic Engineering, Shenzhen University, Shenzhen 518060, China; 3 School of Materials, Sun Yat-Sen University, Shenzhen 518107, China; 4 Center for Composite Materials and Structure, Science and Technology on Advanced Composites in Special Environment Laboratory, Harbin Institute of Technology, Harbin 150080, China; 5 School of Engineering, Hong Kong University of Science and Technology, Hong Kong 999077, China

**Keywords:** vanadium dioxide thin film, finite element simulation, thermal stress, interlayer, phase transition

## Abstract

The buckling, de-lamination, and cracking of the thin film/substrate system caused by thermal stress is the main obstacle for functional failure. Moreover, the thermal stress of vanadium dioxide (VO_2_) thin film may be more complicated due to the stress re-distribution caused by phase transition. Therefore, the thermal stress of VO_2_ thin films deposited on four substrates with different materials (fused silica, silicon slice, sapphire, and glass) has been studied by finite element method in the present work. The influences of external temperature, substrate, and interlayer on thermal stress were analyzed. It was found that the substrates can greatly affect the thermal stresses, which were mainly caused by the mismatch of coefficient of thermal expansion (CTE). The thermal stress had a linear relationship with the external temperature, but this tendency would be redistributed or even change direction when phase transition occurred. The simulated results were in tandem with the analytical method. Meanwhile, the radial stress and shear stress distribution under the influence of phase transition were calculated. In addition, the reduction of thermal stress and shear stress showed that the appropriate interlayer can enhance the adhesive strength effectively.

## 1. Introduction

Vanadium dioxide (VO_2_) undergoes a first-order insulator-to-metal transition from monoclinic phase (M) to rutile phase (R) at around 68 °C, with a drastic change in electric, optical, and other properties [1,2]. It has the potential to be widely used in industrial devices, such as actuators [3], optical switching devices [4,5], and smart windows [6] due to its outstanding physical properties and low phase transition temperature. At present, the research on VO_2_ focuses on how to reduce the phase transition temperature, explain the phase transition mechanism, and optimize the physical parameters (such as emissivity, transmittance, etc.) [1]. However, VO_2_ is mostly applied in the form of a thin film bonded to the substrate in practical products. One of the main obstacles that may lead to functional failure is the residual stress, which may cause significant changes in shape or even produce lamination, buckling, and cracks [7].

The residual stress consists of growth stress, interface stress, and thermal stress, which is mainly produced by the grain growth behavior, lattice mismatch, or chemical reactions [8,9]. Many works have discussed the influence on growth stress and interface stress of different parameters, such as deposition temperature [10], oxygen partial pressure [11], and deposition rate [12]. Unfortunately, the effect of thermal stress was usually ignored. However, thermal stress is common in thin film deposition. In fact, factors such as coefficient of thermal expansion (CTE), substrate, film thickness, and thermal conductivity significantly affect the thermal stress [13]. It is evident that sometimes the thermal stress would also be significant (as high as 20–25% of the total residual stress) if the mismatch of CTE is high [14]. Bielawski [15] measured the residual stress generated in sputter-deposited TiN coatings on Si and steel substrates. They showed that the mechanical properties of the PVD-deposited TiN coating are strongly influenced by the thermal stress in the coatings. Chawla [16] investigated the thermal stress generated in sputter-deposited TiN coating on glass and Si substrates. It was found that the thermal stress induced in the coatings for the rough substrate is higher as compared to that of the planar substrate. The adhesive strength of the TiN coating on the Si substrate is higher when compared to the glass substrate due to the high compressive stress. Tian [17] simulated thermal stress in ion-beam-sputtered Ta_2_O_5_/SiO_2_ multilayer coatings on different substrates. Depending upon analytical results and experimental phenomena, the thermal stress induced from the mismatch of CTE between substrate and coatings seriously affected the performance and longevity of multilayer coatings. Zhang [18] studied the thermal residual stresses in functionally and compositionally graded NiCrAlY/ZrO_2_Y_2_O_3_ TBCs. For functionally graded coatings, the magnitude and distribution of thermal stresses within a graded coating can be adjusted by controlling the compositional gradient, which is characterized by a gradient exponent. According to the literature, the thermal stress simulation of VO_2_ thin film has not yet been found [19]. In the thermal stability test of VO_x_-based thin films, Zhan [20] pointed out that the heat treatment process will cause volume shrinkage, which may lead to tensile stress in the interface. When the tensile stress exceeds the limit condition, micro-cracks occur. Furthermore, when phase transition occurs in the VO_2_, it causes the lattice to expand by 1% along the c_R_-axis. Meanwhile, the lattice shrinks by 0.6% and 0.1% along the tetragonal a_R_-axis and b_R_-axis [21], respectively. The high bending caused by phase transition can even be applied to thermal-drive actuators [22]. Therefore, it is very meaningful to quantify the thermal stress in various applications, which is also essential for understanding the de-lamination and cracking of thin films.

In this paper, thermal stress in VO_2_ thin film was successfully simulated using a finite element method. The influence of the temperature, interlayer, and substrate on thermal stress was discussed. The purpose of this work is to find out the control parameters through the simulation so as to optimize the design of a high-quality VO_2_ thin film/substrate system.

## 2. Analytical Model

The analytical model proposed by Tsui and Clyne [23] was used to predict residual stress in progressively deposited thin film systems for the planar geometry configuration. By combining the model and Stoney equation [17], the equation for thermal stress in thin films can be obtained as:

(1) $$\sigma =\frac{E_{ef}\int_{T_{r}}^{T_{d}}(\alpha_{s}-\alpha_{f})dT}{1+4(\frac{E_{ef}}{E_{es}})(\frac{h}{H})}$$

where $E_{ef}=E_{f}/(1-\upsilon_{f})$, $E_{es}=E_{s}/(1-\upsilon_{s})$, $E_{f}$, $E_{s}$, $\upsilon_{f}$, $\upsilon_{s}$, $T_{r}$, $T_{d}$, h, H, $\alpha_{f}$ and $\alpha_{s}$ are the effective elastic modulus of the thin film, effective elastic modulus of the substrate, elastic modulus of the thin film, elastic modulus of the substrate, Poisson’s ratio of the thin film, Poisson’s ratio of the substrate, room temperature, external temperature, thin film thickness, substrate thickness, and CTE of the thin film and substrate, respectively.

It is worth noting that in the initial stage of film deposition, stress relaxation sometimes occurs near the interface, which may be caused by structural defects, and stress relaxation increases with the increase of film thickness. Stress relaxation may lead to nanoscale line cracks which affect transport properties [24]. Similarly, interface defects and lattice mismatch accompany the initial stage of film deposition because the thin film and the substrates are not perfectly bonded in practice [25,26]. However, previous work has shown that when this kind of thickness rose to an affirmatory value, the interface stress did not play a crucial part in the entire residual stress, and so the effect of stress relaxation, interface defects, and lattice mismatch can be ignored [25]. Therefore, this analytical model is not applicable to the thermal stress calculation of ultra-thin films.

## 3. Finite Element Model

**Figure 1:**
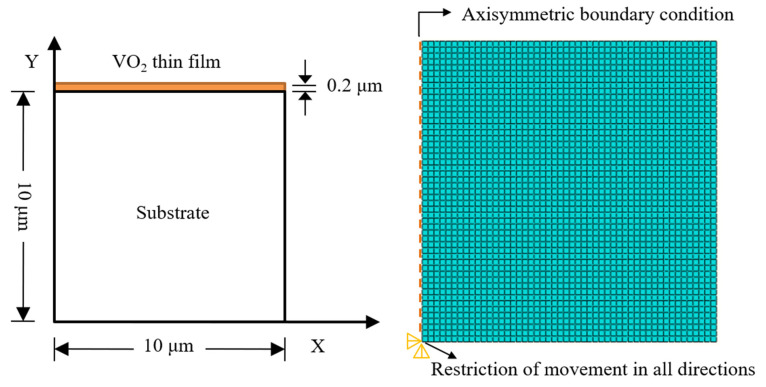
Boundary conditions and finite element meshes diagram of the finite element model.

A numerical simulation of thermal stress on VO_2_ thin film under different conditions was calculated using ABAQUS finite element analysis software. In order to simplify the calculation, the axis symmetry option was considered in the present work, as shown in Figure 1. Furthermore, the diameter of the cylindrical substrate and the thickness of the substrate and thin film were fixed at 20 μm, 10 μm, and 0.2 μm, respectively. It was modeled using the four-node bilinear axisymmetric quadrilateral element CAX4R. Mapped meshing with a quadrilateral-shaped element was used to mesh the model. Fine meshing was also performed near the thin film/substrate interface because this area was under a very high stress concentration. The left side of the model used as the symmetric axis was restricted at the *x* axis (*x* = 0, U1 = 0), and the vertical displacement was constrained at the bottom left coordinate origin (*x* = 0, *y* = 0, U1 = U2 = UR3 = 0). All other edges were free so that bending was permitted to take place when the temperature changed. The thermal stress in the film/substrate system would be produced by loading the uniform temperature load on the model. The thermal stresses in the FEM calculation were computed as maximum Von Mises stress in the thin film.

Several assumptions were made for simplicity of calculations: (a) the thin film, interlayer, and substrate materials were both isotropic and linearly elastic; (b) the thin film and substrates were perfectly bonded at the interface; (c) a uniform temperature was established in the body; (d) there was no initial residual stress; (e) the temperature dependence of CTE was negligible except for VO_2_. The physical and thermal properties of all materials for thin films and substrates used in the simulation are listed in Table 1 [16,27,28,29,30,31]. Due to its crystallographic nature, VO_2_ is monoclinic below 68 °C and rutile above 68 °C, while the substrates and interlayers are in the amorphous phase except for sapphire and TiO_2_.

## 4. Results and Discussion

### 4.1. Effect of Temperature and Substrates

**Figure 2:**
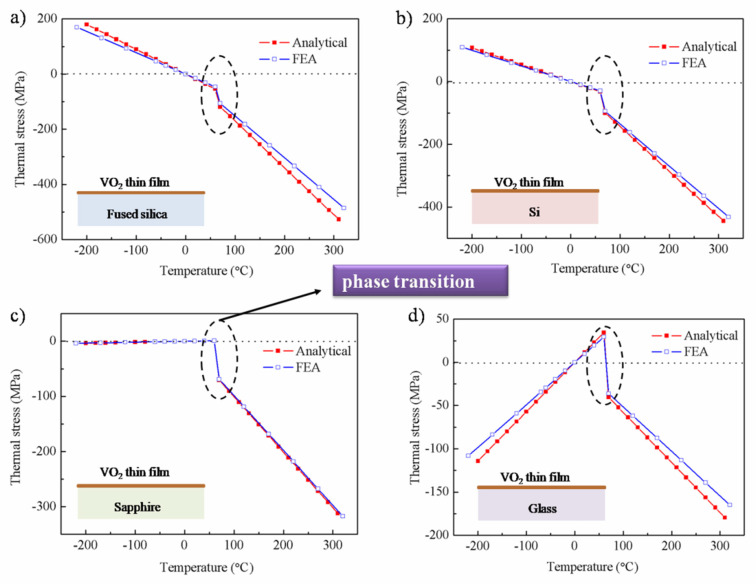
The thermal stress of VO_2_ thin film deposited on four substrates: (**a**) fused silica, (**b**) Si, (**c**) sapphire, (**d**) glass.

According to Equation (1), the influence of thickness can be ignored when the ratio of the film thickness to the substrate thickness is small. By substituting the temperature (−200–300 °C) and the properties of the substrates in Equation (1), the variation of thermal stress generated in the VO_2_ thin film deposited on the four substrates is shown in Figure 2.

Firstly, it can be seen that the thermal stress changes linearly with the temperature under the four substrates, and the simulation results fit well with the theoretical model. Although there is a certain deviation between the curves, the error remains within 5%. These verified the accuracy of the simulation model. At the same time, this monotonic linear relationship is due to the mismatch of CTE between the film and the different substrates. More strikingly, when the temperature exceeds approx. 68 °C, there is an obvious sudden change in thermal stress due to the phase transition. This is because the phase transition will lead to a change in CTE and the elastic modulus, resulting in the re-distribution of thermal stress.

Then, for the fused silica and Si substrate, the thermal stress has a “tensile–compressive stress conversion” when the temperature is zero. When the phase transition occurred, the growth trend of thermal stress was exacerbated. When the temperature is fixed at 300 °C, the stress of the thin film deposited on the fused silica substrate is the largest, up to 485.6 MPa. Interestingly, the thermal stress presents differently for the sapphire and glass substrates. For the sapphire substrate, the thermal stress is close to zero until the phase transition occurred, which is due to their virtually identical CTE. However, it suddenly changed into compressive stress, accompanied by the CTE of VO_2_, from 5.7 × 10^−6^/°C to 13.35 × 10^−6^/°C after the phase transition, as listed in Table 1. Specially, for the glass substrate, the behavior of thermal stress is more complex. As the temperature goes from low to high, the thermal stress showed a phenomenon of “compressive–tensile–compressive stress conversion”. This is also caused by the mismatch of CTE. It is worth noting that the multi-stress conversion is disadvantageous in the environment of a cold–hot cycle. Therefore, the phase transition could affect the trend of thermal stress, even the stress direction, and reasonable selection of substrate materials can reduce the impact of thermal stress.

### 4.2. Distribution of Thermal Stress

According to Suhir’s theory [26], in light of the impact on thermal stress by substrates, the distribution of radial stress and shear stress was studied by measuring an environment with hot and cold temperatures (±200 °C).

**Figure 3:**
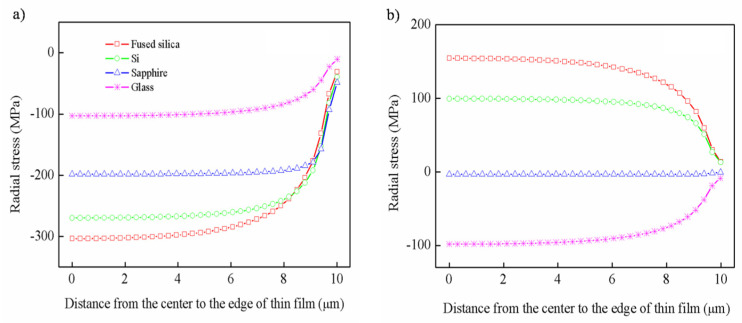
The distribution of radial stress along the x direction: (**a**) hot temperature, (**b**) cold temperature.

As shown in Figure 3a, the radial stresses along the x direction are all compressive stresses, which are caused by the fact that the CTE of VO_2_ is greater than that of the four substrates after the phase transition. The radial stress decreases exponentially with the increase of the distance from the center and approaches zero at the edge of the film. Among them, the radial stress of the thin film on the fused silica substrate is the largest, up to −303.51 MPa, about three times than that of the glass substrate. However, when the VO_2_ thin film is at a cold temperature, although the radial stress also shows exponential distribution, the stress direction for the different substrates is not consistent, as shown in Figure 3b. The radial stress of the thin films is tensile stress on the fused silica and Si substrates, compressive stress on the glass substrate, and close to zero on the sapphire substrate.

**Figure 4:**
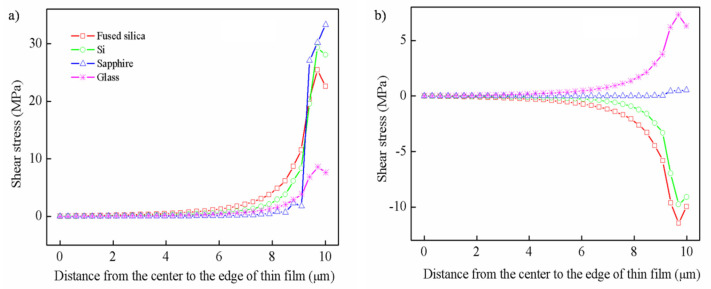
The distribution of shear stress along the x direction: (**a**) hot temperature, (**b**) cold temperature.

It is necessary to study the shear stress because if the stress along the interface exceeds the bond strength between the thin film and substrate, this would cause de-adhesion [32,33]. Contrary to the radial stress, the shear stress along the x direction is all tensile stress at the hot temperature, as shown in Figure 4a. As a result of stress concentration, the shear stress rapidly increases near the edge region and is basically zero in the range of 8μm from the center. These results conform to the theoretical model and are also similar to other studies [33]. Among them, the shear stress of the thin film deposited on the glass substrate is obviously small, close to 7.62 MPa, while the other three substrates are virtually the same. Similarly, the distribution of shear stress still changes rapidly near the edge at the cold temperature, as shown in Figure 4b. Contrary to the radial stress, the shear stress of the thin films is compressive stress on fused silica and Si substrates, tensile stress on the glass substrate, and nearly zero on the sapphire substrate.

**Figure 5:**
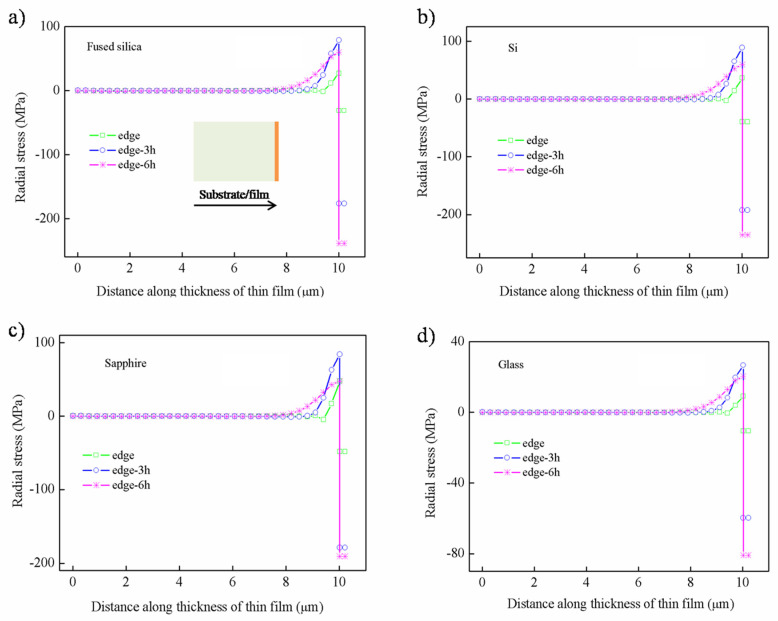
The distribution of radial stress along the thickness at hot temperature: (**a**) fused silica, (**b**) Si, (**c**) sapphire, (**d**) glass.

The distribution of the radial stress at the hot temperature along the thickness is shown in Figure 5. It can be seen that the distribution of radial stress is mainly concentrated approx. 8 μm away from the bottom, and the maximum value occurs at the junction of the thin film and the substrate, accompanied by sudden “tensile–compressive stress conversion”. Among them, the stress of the thin film deposited on the glass substrate is clearly smaller than that of the other three substrates. Similarly, the radial stress distribution at the cold temperature can be seen in the supporting information (Figure S1).

**Figure 6:**
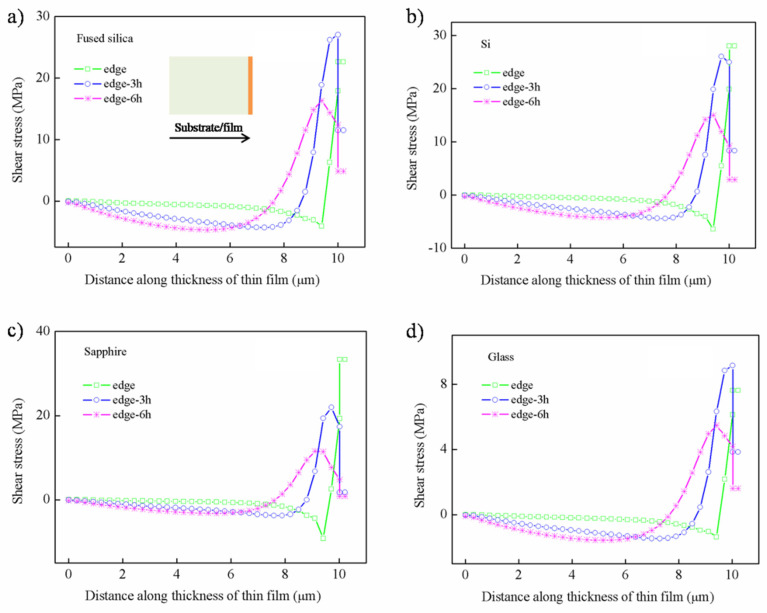
The distribution of shear stress along the thickness at hot temperature: (**a**) fused silica, (**b**) Si, (**c**) sapphire, (**d**) glass.

The shear stress along the thickness at different positions from the edge to the center is plotted in Figure 6. The stress distribution under the four substrates is virtually the same at the hot temperature. It can be concluded that the shear stress reversals from compressive to tensile stress occurs approx. 8 μm from the bottom, and the maximum tensile stress is observed at the interface.

The compressive shear stress is observed in the range of approx. 8μm, and it increases with the distance, such as 0 h (h is the thickness of thin film), 3 h, and 6 h away from the edges. On the contrary, the tensile shear stress shows a decreasing trend upon moving away 0 h, 3 h, and 6 h from the edge. Specially, the shear stress on the edge shows a slightly different trend, which may be caused by stress release. It can be seen that the shear stress on the glass substrate is obviously the smallest, while the others are basically the same. Similarly, the shear stress distribution at the cold temperature can be seen in the supporting information (Figure S2).

Research shows that, whether the stress is along the x direction or along the thickness, the substrate has little effect on the stress distribution but has a clear impact on the stress value. In particular, it should be noted that phase transition can affect the direction of stress due to the secondary mismatch of CTE.

### 4.3. Effect of Interlayer

In practice, buffer layers, or the so-called interlayer, are sometimes introduced in VO_2_ thin films to optimize their physical properties [34,35]. Therefore, it is necessary to study the influence of the interlayer on thermal stress. First, we fixed fused silica as the substrate. At the same time, the film thickness was maintained at 200 nm, and the interlayer thickness was set to 50 nm in the geometric model. TiO_2_ and SiO_2_ are often used as an interlayer because of their excellent optical properties [36,37,38]. Meanwhile, the CTE of SiO_2_ is close to the substrate, and the CTE of TiO_2_ is close to the VO_2_ thin film. Therefore, these two materials were selected to investigate the influence of the interlayer by simulation.

**Figure 7:**
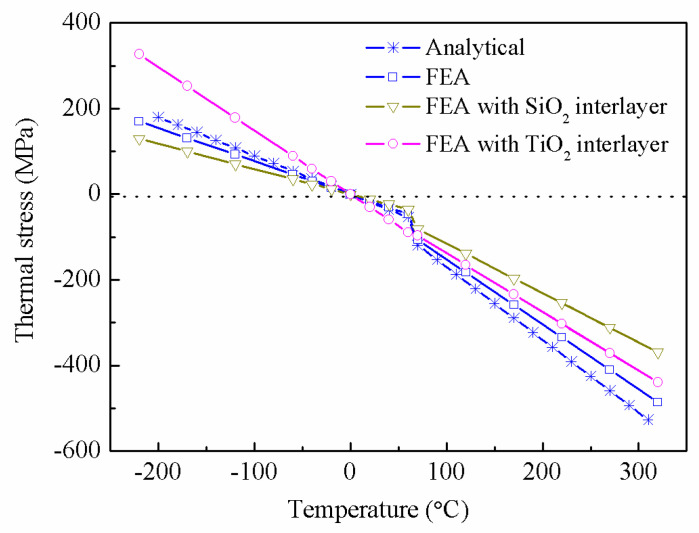
The distribution of thermal stress with the introduction of an interlayer.

The simulated results as shown in Figure 7 indicate that the introduction of two kinds of interlayer can change the rate of thermal stress, but the influence is different at a hot temperature or cold temperature. When the temperature is 300 °C, the maximum thermal stress changes from −485.6 MPa to −368.7 Mpa with the introduction of the SiO_2_ interlayer. These show that the thermal stress can be reduced by introducing an interlayer in hot temperatures. Moreover, the effect of SiO_2_ on relieving thermal stress is better than TiO_2_. Similarly, the SiO_2_ interlayer can also reduce the thermal stress at cold temperatures. However, it is worth noting that TiO_2_ has the opposite effect.

**Figure 8:**
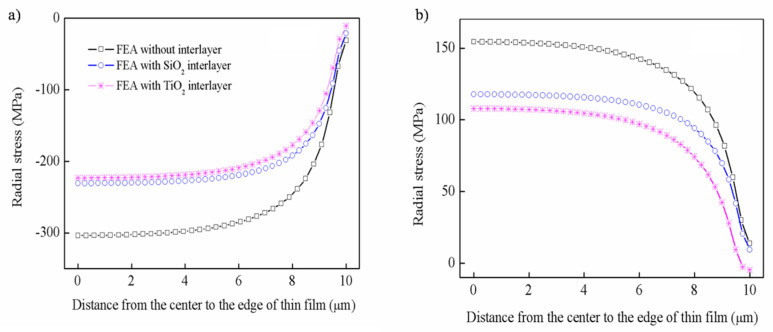
The distribution of radial stress along the x direction with the introduction of an interlayer: (**a**) hot temperature, (**b**) cold temperature.

As in Section 4.2, the effect of the interlayer on stress distribution was studied. The distribution of radial stress along the x direction with the introduction of the interlayer is shown in Figure 8. It can be seen that whether at a hot or cold temperature, these two kinds of interlayer can significantly reduce the stress, and the extent of this effect is almost the same. The radial stress decreases from −303.51 MPa to −222.98 MPa in the central area of the film at hot temperatures, and the radial stress is reduced from 154.41 MPa to 110.73 MPa at cold temperatures, respectively.

**Figure 9:**
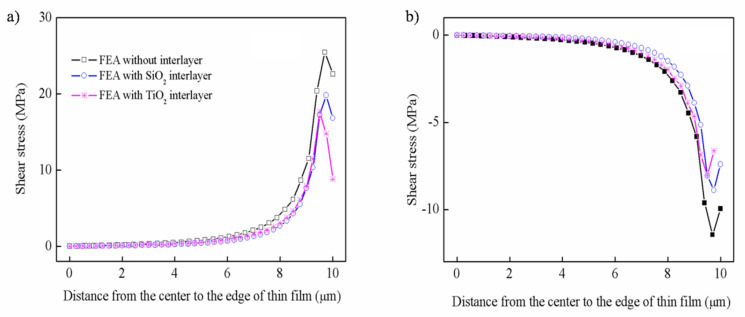
The distribution of shear stress along the x direction with the introduction of an interlayer: (**a**) hot temperature, (**b**) cold temperature.

The distribution of shear stress along the x direction with the introduction of the interlayer is shown in Figure 9. Similar to radial stress, it can be seen that whether at a hot or cold temperature, the interlayer can significantly alleviate the shear stress at the edge of the thin film. The shear stress was reduced from 22.62 MPa to 8.81 MPa at the hot temperature and from −9.96 MPa to −6.63 MPa at the cold temperature, respectively.

**Figure 10:**
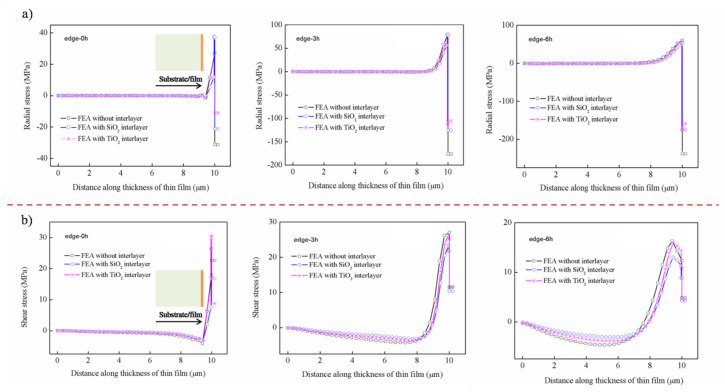
The distribution of thermal stress along the thickness with the introduction of an interlayer at hot temperature: (**a**) radial stress, (**b**) shear stress.

Besides that, the radial stress and shear stress along the thickness at different positions from edge to center with the introduction of the interlayer is shown in Figure 10. It can be seen that the interlayer can reduce the radial stress of the film edge at hot temperatures, and the farther away from the edge, the better the effect, as shown in Figure 10a. Unlike the radial stress, the two kinds of interlayer have little effect on the shear stress of the edge; however, it shows a relief effect on the interface, as shown in Figure 10b. Similarly, the influence of the interlayer on the radial stress and shear stress along the thickness at cold temperature can be seen in the supporting information (Figure S3).

Mechanically, both SiO_2_ as a flexible interlayer and TiO_2_ as a rigid interlayer can alleviate crack propagation at the interface, resulting in the reduction of shear stress. The smooth transition of CTE from the substrate to the VO_2_ thin film can be considered an effective explanation for the reduction of stress. Furthermore, theoretically, in terms of crystallography, TiO_2_ is considered to help achieve the degree of crystallinity for the later-deposited VO_2_ thin films. For example, Cho [36] proposed that the interface between VO_2_ and TiO_2_ dominates the orientation of crystallography with strain stabilization. Ding [37] showed TiO_2_ to be a suitable buffer layer, as it can reduce the lattice strain along the c-axis for high-performance thin film. Similarly, SiO_2_ has an effect on the particle size and morphology of VO_2_ films, which is conducive to the uniformity of grains and reduces the agglomeration degree of VO_2_ grains. In addition, the existence of the amorphous structure of SiO_2_ among VO_2_ crystalline lattices reduces the asymmetric shear stress in cooling [38]. Therefore, these factors may also play an important role in relieving thermal stress. According to the obtained results, the reasonable use of an interlayer is conducive to relieving thermal stress. More importantly, it can reduce the shear stress at the interface and improve the adhesion between the thin film and the substrate.

## 5. Conclusions

In this study, the thermal stress of VO_2_ thin film deposited on four substrates (fused silica, silicon slice, sapphire, and glass) was simulated by ABAQUS. The simulation results have been compared with a theoretical model to show rationality and feasibility. Thermal stress is seriously affected by the substrates because of the mismatch in CTE. It is worth noting that although thermal stress exhibits a linear relationship with temperature, when the temperature exceeds approx. 68 °C, there is a noticeable sudden change due to the phase transition, even leading to the “compression–tensile stress conversion”. Whether at hot or cold temperatures, the radial stress along the x direction changes exponentially with the increase of distance from the center and approaches zero at the edge. However, the distribution of shear stress is the opposite, and the highest shear stress occurs at the edge, which is caused by stress concentration. Similarly, the radial stress along the thickness under the four substrates is mainly concentrated approx. 8μm away from the bottom, and the maximum stress appears at the interface, accompanied by sudden “tensile–compressive stress conversion”. Also, the shear stress reversal occurs approx. 8μm from the bottom, and the maximum stress is observed at the interface, respectively. The appropriate interlayer not only can greatly reduce the thermal stress, but also effectively enhance the adhesive strength of the interface in the film/substrate system.

## Figures and Tables

**Table 1:** The detailed parameters used in simulation.

Materials	Elastic Modulus (GPa)	Passion’s Ratio	CTE (10^−6^/°C)
VO_2_ [27]	130 (0~68 °C) 100 (>68 °C)	0.25	5.7 (0~68 °C) 13.35 (>68 °C)
Fused silica [28,29]	73.1	0.17	0.5
Si [28,29]	131	0.28	2.6
Sapphire [30]	503	0.29	5.8
Glass [16]	69	0.24	9
TiO_2_ [31]	175.61	0.36	8.5
SiO_2_ [28,29]	73.1	0.17	0.55

## Data Availability

The raw data required to reproduce these findings are available on request.

## Supplementary Materials

The following supporting information can be downloaded at: https://www.mdpi.com/article/10.3390/nano12234262/s1, Figure S1. The distribution of radial stress along the thickness at cold temperature: (a) Fused silica, (b) Si, (c) Sapphire, (d) Glass. Figure S2. The distribution of shear stress along the thickness at cold temperature: (a) Fused silica, (b) Si, (c) Sapphire, (d) Glass. Figure S3. The distribution of thermal stress along the thickness with the introduction of interlayer at cold temperature: (a) radial stress, (b) shear stress.
