# Supplementary material for: Analysis of Thermal Stress in Vanadium Dioxide Thin Films by Finite Element Method
Source: Nanomaterials (Basel). 2022 Nov 30;12(23):4262. doi: 10.3390/nano12234262 (PMC9735821; doi:10.3390/nano12234262)
Supplement: Supplementary file 1 [file nanomaterials-12-04262-s001.zip › nanomaterials-1984937-supplementary.pdf]

nanomaterials

MDPI

Supplementary Materials

Analysis of Thermal Stress in Vanadium Dioxide Thin Films by Finite Element Method

Yuemin Wang [1,2], Lebin Wang [3], Jinxin Gu [4], Xiangqiao Yan [4], Jiarui Lu [5], Shuliang Dou [4,*], Yao Li [4], and Lei Wang [1,*]

[1] Shenzhen Key Laboratory of Polymer Science and Technology, College of Materials Science and Engineering, Shenzhen University, Shenzhen 518060, China; wangyuemin@szu.edu.cn
[2] College of Physics and Optoelectronic Engineering, Shenzhen University, Shenzhen 518060, China
[3] School of Materials, Sun Yat-Sen University, Shenzhen 518107, China; wanglb33@mail.sysu.edu.cn
[4] Center for Composite Materials and Structure, Science and Technology on Advanced Composites in Special Environment Laboratory, Harbin Institute of Technology, Harbin 150080, China; 19b925079@stu.hit.edu.cn (J.G.); yanxiangqiao406@163.com (X.Y.)
[5] School of engineering, Hong Kong University of Science and Technology, Hong Kong 999077, China; jlubs@connect.ust.hk
* Correspondence: dousl@hit.edu.cn (S.D.); wl@szu.edu.cn (L.W.)

The distribution of the radial stress at the cold temperature along the thickness is shown in Figure S1. It can be seen that the radial stress is also mainly concentrated in ~8 μm away the bottom, and the maximum stress occurs at the junction, which is similar to at hot temperature. However, the difference is that the radial stress of the thin film deposited on the sapphire substrate is obvious smaller than others. Moreover, the sudden "tensile-compressive stress conversion" of the film deposited on sapphire and glass substrate is opposite to others.

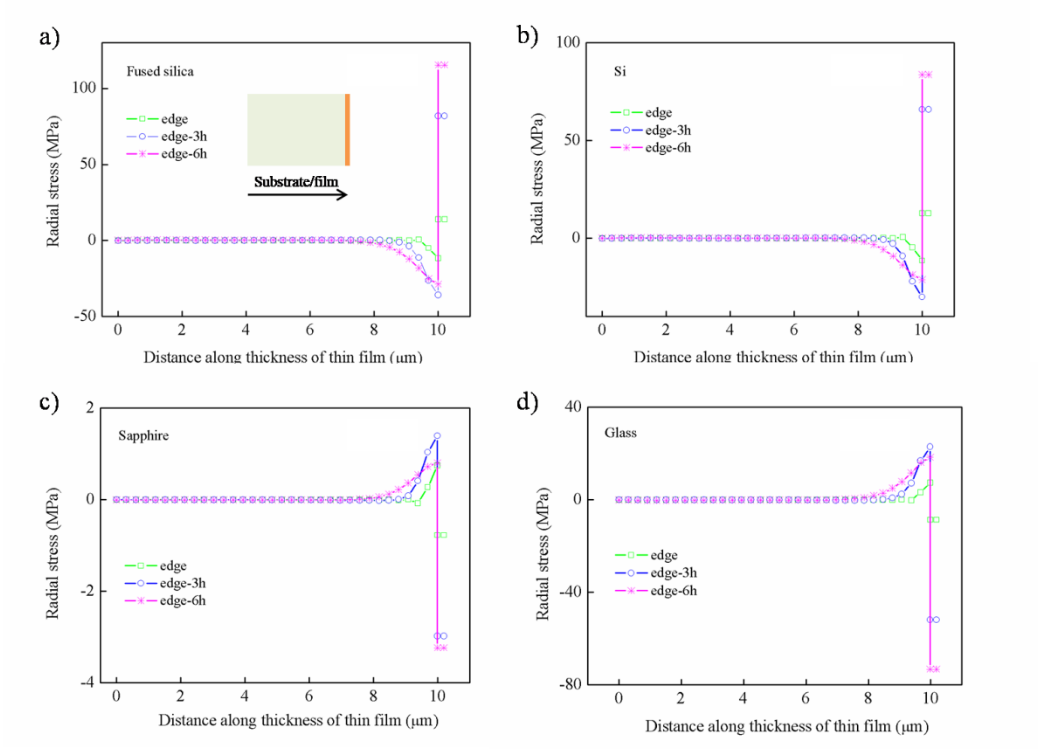

Figure S1. The distribution of radial stress along the thickness at cold temperature: (**a**) Fused silica, (**b**) Si, (**c**) Sapphire, (**d**) Glass.

The shear stress at cold temperature along the thickness at different position from the edge to the center is plotted in Figure S2. Opposite to the distribution at hot temperature, for fused silica and Si substrate, the shear stress reversals from tensile to compressive stress occurs in ~8 μm from its bottom and the maximum compressive stress is observed at the interface. Moreover, the shear stress on the sapphire substrate is the smallest.

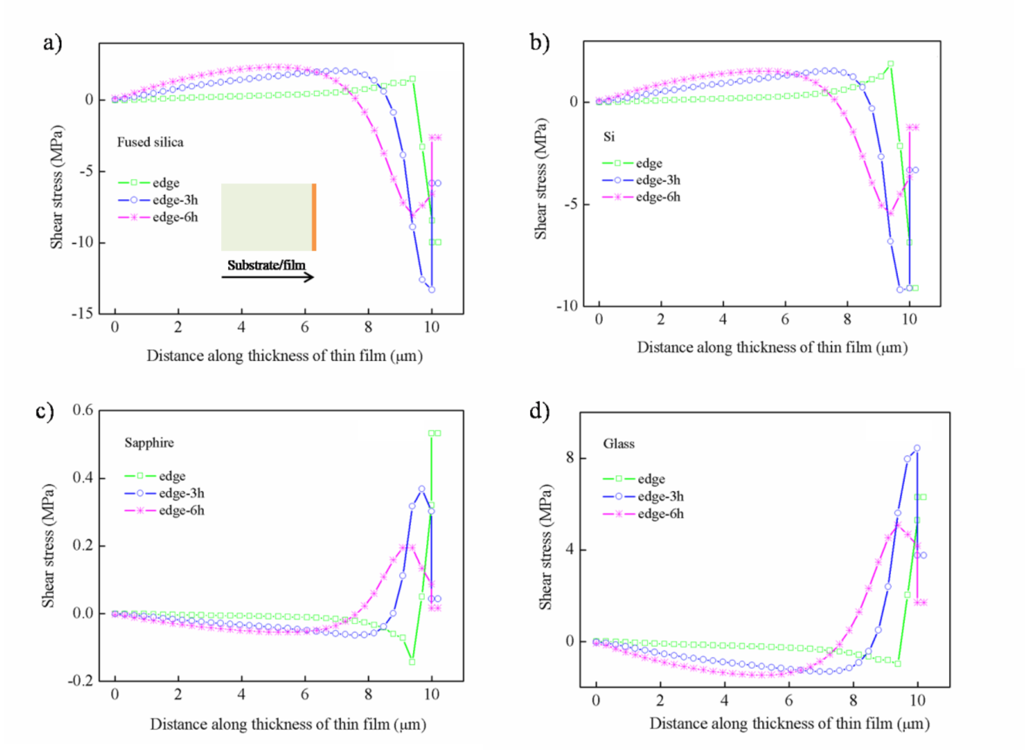

Figure S2. The distribution of shear stress along the thickness at cold temperature: (**a**) Fused silica, (**b**) Si, (**c**) Sapphire, (**d**) Glass.

The radial stress and shear stress at cold temperature along the thickness at different position from edge to center with the introduction of interlayer is shown in Figure S3. Similarly, the interlayer can reduce the radial stress of the film edge, and the farther away from the edge, the better the effect. Unlike the radial stress, the two kinds of interlayer have little effect on the shear stress of the edge; however, it shows a relief effect on the interface.

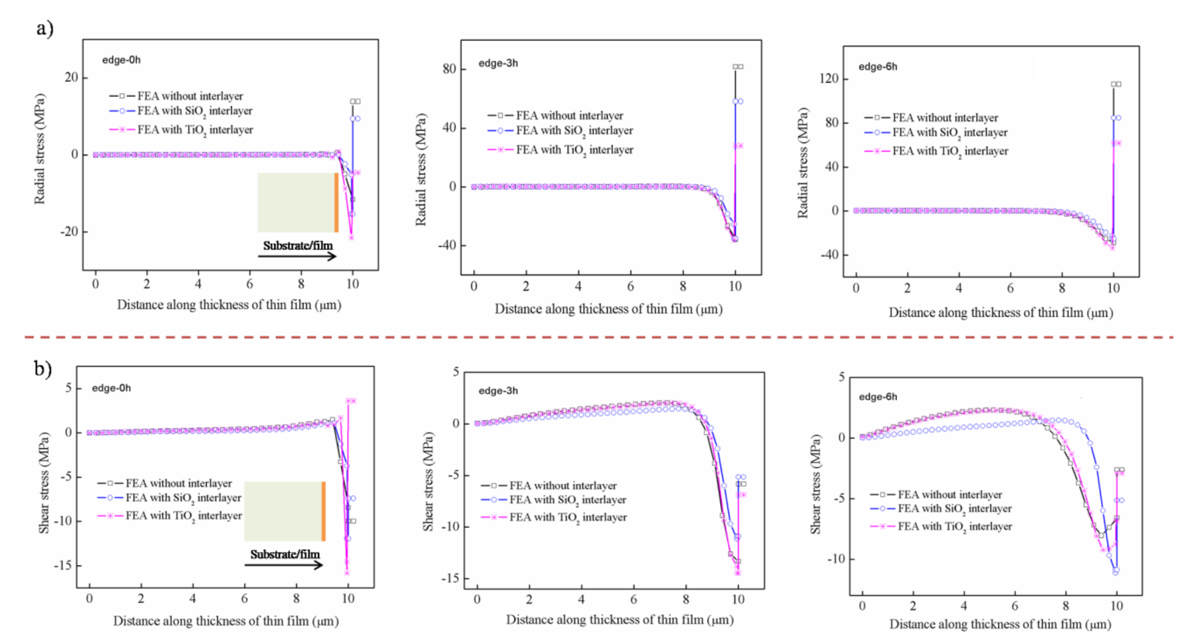

Figure S3. The distribution of thermal stress along the thickness with the introduction of interlayer at cold temperature: (**a**) radial stress, (**b**) shear stress.
